# Supplementary material for: Reactive Oxygen Species Drive the Aberrant Immune Response to a C. neoformans Chitin Synthase 3 (chs3Δ) Mutant
Source: bioRxiv. 2025 Jun 27:2025.06.24.661434. Preprint. [Version 1] doi: 10.1101/2025.06.24.661434 (PMC12262250; doi:10.1101/2025.06.24.661434)
Supplement: 1 [file NIHPP2025.06.24.661434V1-supplement-1.pdf]

bioRxiv preprint doi: https://doi.org/10.1101/2025.06.24.661434; this version posted June 27, 2025. The copyright holder for this preprint (which was not certified by peer review) is the author/funder, who has granted bioRxiv a license to display the preprint in perpetuity. It is made available under aCC-BY 4.0 International license.

Table S1. Antibodies for flow analysis.

Antigen	Clone	Fluorophore	Dilution	Company
CD3	17-A2	eFluor506	1:250	Invitrogen
CD11b	M1/70	PE-Cy7	1:125	BioLegend
CD11c	HL3	PE-CF594	1:125	BD Biosciences
CD16/CD32 Fc Block	2.4G2	(not applicable)	1:500	BD Biosciences
CD19	1D3	eFluor506	1:500	Invitrogen
CD24	M1/69	BV421	1:250	BioLegend
CD45	30-F11	PerCP	1:250	BioLegend
CD64	X54-5/7.1	BV605	1:75	BioLegend
CD103	2E7	APC-Cy7	1:125	BioLegend
F4/80	BM8	Alexa647	1:125	BioLegend
Ly6C	HK1.4	BV650	1:250	BioLegend
Ly6G	1A8	BV785	1:250	BioLegend
Siglec-F	S17007L	PE	1:250	BioLegend

bioRxiv preprint doi: https://doi.org/10.1101/2025.06.24.661434; this version posted June 27, 2025. The copyright holder for this preprint (which was not certified by peer review) is the author/funder, who has granted bioRxiv a license to display the preprint in perpetuity. It is made available under aCC-BY 4.0 International license.

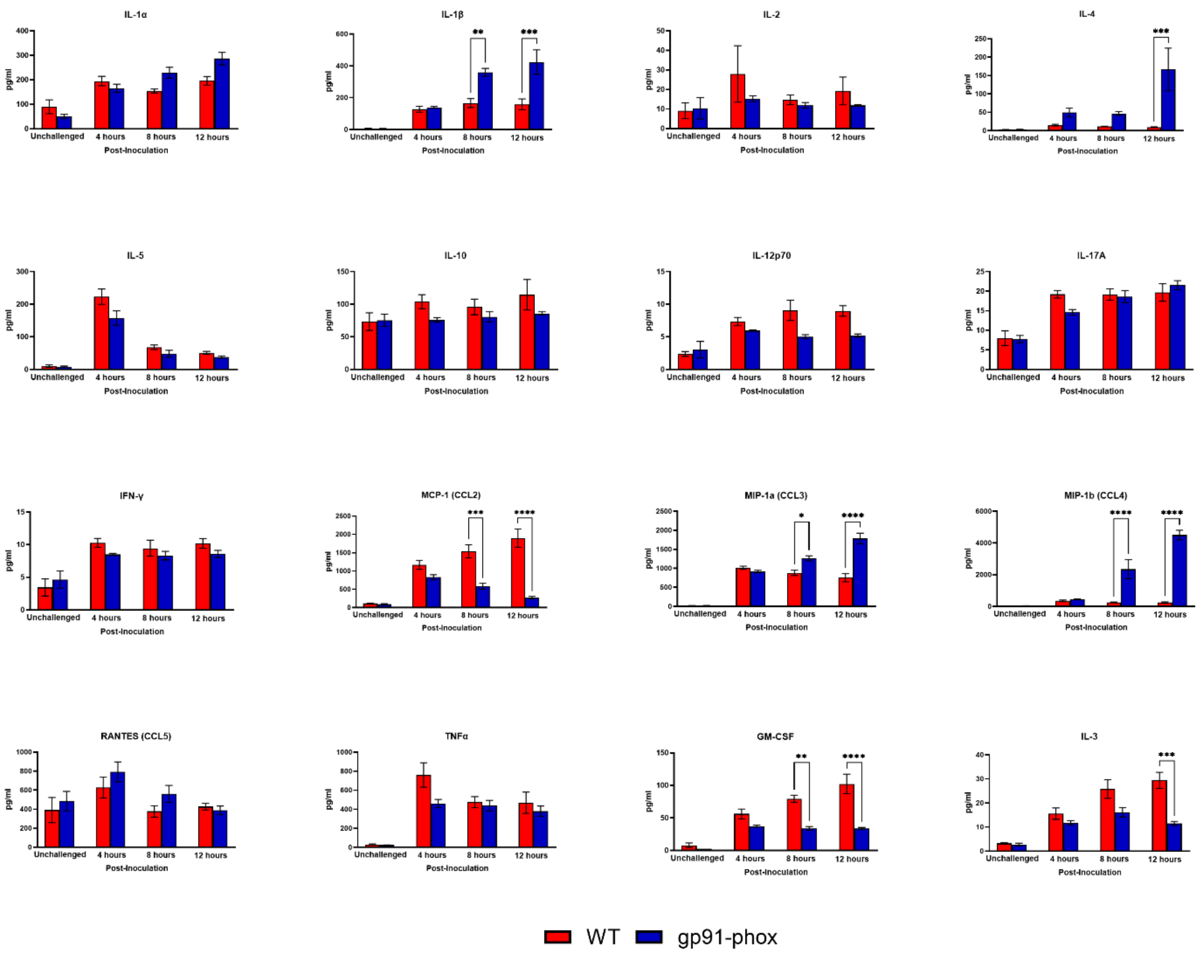

Supplementary Figure 1. Cytokine/chemokine analysis: Male and female C57BL/6 or gp91^{phox-} mice were inoculated with 10^7 CFUs of HK *chs3*Δ. Homogenates were prepared from the lungs of each group at the indicated time point as well as a phosphate-buffered saline (PBS) control for each group. Cytokine/chemokine responses were determined from the lung homogenates. Data are cumulative of two experiments for a total of 8 mice per group per timepoint. Values are means ± standard errors of the means (SEM). (*, *P* <0.05, **, *P* <0.005, ***, *P* <0.001).

bioRxiv preprint doi: https://doi.org/10.1101/2025.06.24.661434; this version posted June 27, 2025. The copyright holder for this preprint (which was not certified by peer review) is the author/funder, who has granted bioRxiv a license to display the preprint in perpetuity. It is made available under aCC-BY 4.0 International license.

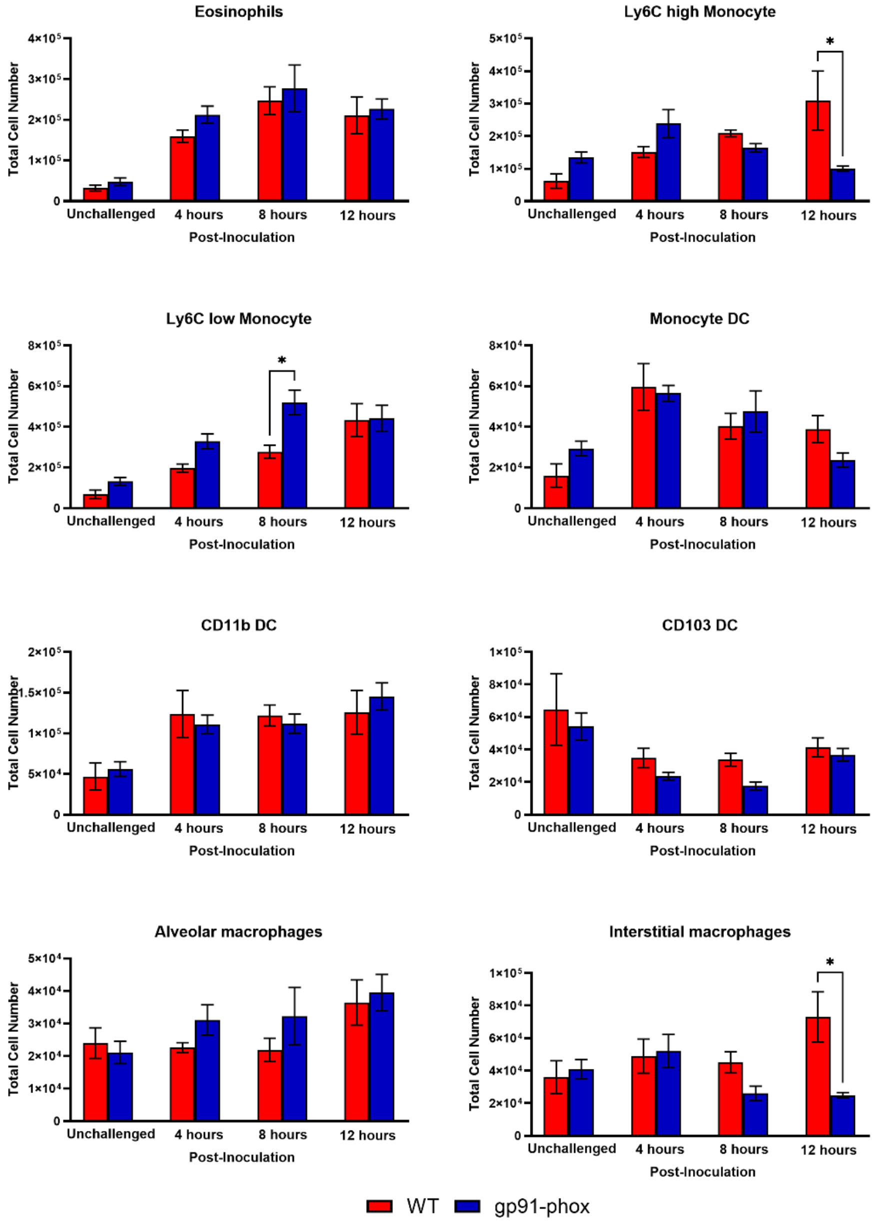

Supplementary Figure 2. Flow cytometry analysis: Male and female C57BL/6 or gp91^{phox-} mice were inoculated with 10^7 CFUs of HK *chs3*Δ. At the indicated time point, pulmonary leukocytes were isolated from the lungs of mice of each group and subjected to flow cytometry analysis. Data are cumulative of two experiments for a total of 8 mice per group per timepoint. Values are means ± standard errors of the means (SEM). (*, *P* <0.05).
